# CEA as a blood-based biomarker in anal cancer

**DOI:** 10.18632/oncotarget.27959

**Published:** 2021-05-25

**Authors:** Robert Hester, Shailesh Advani, Asif Rashid, Emma Holliday, Craig Messick, Prajnan Das, Yi-Qian N. You, Cullen Taniguchi, Eugene J. Koay, Brian Bednarski, Miguel Rodriguez-Bigas, John Skibber, Robert Wolff, George J. Chang, Bruce D. Minsky, Wai Chin Foo, Nicole Rothschild, Van K. Morris, Cathy Eng

**Affiliations:** ^1^Division of Cancer Medicine, The University of Texas - MD Anderson Cancer Center, Houston, TX, USA; ^2^Division of Oncology, Terasaki Foundation of Biomedical Sciences, Los Angeles, CA, USA; ^3^Department of Pathology, The University of Texas - MD Anderson Cancer Center, Houston, TX, USA; ^4^Department of Radiation Oncology, The University of Texas - MD Anderson Cancer Center, Houston, TX, USA; ^5^Department of Surgical Oncology, The University of Texas - MD Anderson Cancer Center, Houston, TX, USA; ^6^Department of Gastrointestinal Medical Oncology, The University of Texas - MD Anderson Cancer Center, Houston, TX, USA; ^7^Department of Hematology/Oncology, Vanderbilt Ingram Cancer Center, Nashville, TN, USA; ^*^These authors contributed equally to this work

**Keywords:** carcinoembryonic antigen, squamous cell carcinoma of anal canal, anal cancer, biomarkers, HPV

## Abstract

Background: The clinical utility of a blood-based biomarker in squamous cell carcinoma of the anus (SCCA) is unknown. We analyzed carcinoembryonic antigen (CEA), a commonly employed assay for patients with colorectal adenocarcinoma, as a serum biomarker for patients with biopsy-proven SCCA.

Materials and Methods: Medical records from 219 patients with biopsy-proven SCCA at the University of Texas MD Anderson Cancer Center were reviewed under an IRB-approved protocol from 2013 to 2020 to assess for correlations between CEA levels and corresponding clinical and pathologic characteristics.

Results: The mean CEA among subgroups by clinical status at the time of presentation to our institution was highest among those patients with metastatic SCCA to visceral organs (M-V, 20.7 ng/mL), however this finding was not statistically significant by ANOVA (*p* = .74). By clinical subgroup, the percentage of patients with an abnormally elevated CEA was highest in those patients with metastatic disease to lymph nodes (M-L, 41.2%) followed by recurrent/unresectable SCCA (36.8%), and metastatic SCCA to visceral organs (M-V, 35.2%), and was statistically significant between groups (Fisher’s exact test *p* = .02). Using RECIST criteria for tumor progression and disease response, the mean change in CEA for patients with progression was an increase in 19 ng/mL, compared to a change of –7.3 ng/mL in those with disease response (*p* = .004). We likewise assessed whether CEA levels were associated with survival outcomes for all patients with metastatic SCCA, and found no correlation between CEA and likelihood for survival in a ROC analysis (multivariate, age-adjusted analysis for CEA cutoff of 8, HR = 1.01, 95% CI 0.52–1.96).

Conclusions: Despite interesting patterns of abnormally high CEA in SCCA patients with advanced disease, and correlation of increased CEA with disease progression (and conversely decreased CEA with disease response), CEA is not associated with survival outcomes in SCCA, and is not a clinically relevant biomarker in this disease.

## INTRODUCTION

Squamous cell carcinoma of the anal canal (SCCA) is a rare cancer of the anogenital track with an estimated incidence of about 8500 new cases and 1350 deaths in 2020 in the U.S. annually, comprising 2–3% of all gastrointestinal malignancies [1]. Women are more commonly diagnosed than men with an approximate 2:1 incidence. The development of SCCA is closely associated with prior HPV infection (predominantly HPV-16) [2]. While patients with localized disease may be curatively treated with concurrent chemoradiation [3, 4], surgery remains an effective option for patients with recurrent or persistent disease following chemoradiation [5]. Patients with metastatic disease are generally treated with doublet cytotoxic chemotherapy backbones based upon platinum/taxane or fluoropyrimidine/platinum [6]. Recently, systemic responses to anti-PD-1 monotherapy have been reported for patients with chemotherapy refractory to metastatic SCCA [7, 8].

Routine, readily available blood-based markers are often utilized in the clinical management of patients with solid tumors across a variety of clinical settings. For example, trends in biomarkers such as carcinoembryonic antigen (CEA), carbohydrate antigen (CA) 19-9, prostate-specific antigen (PSA) and carbohydrate antigen (CA)125 can be monitored serially over time for patents with colorectal cancer, pancreatic cancer, prostate cancer, and ovarian cancer, respectively, as a surrogate for changes in amount of tumor present [9]. Use of circulating tumor DNA (ctDNA) for tracking HPV ctDNA as a response to therapy for SCCA has shown promise as a tumor-specific blood-based biomarker in small series, but thus far its use is limited to the research setting [10, 11]. To date, no blood-based biomarker for tracking responses to HPV-associated cancers is readily available to clinical oncologists for routine use.

Among anal cancer patients, one series examined 106 patients with early-stage SCCA treated definitely with chemoradiation and did not find clinical utility in the measurement of CEA in this subset of patients with anal cancer [12]. Since no blood-based biomarkers are currently available in a CLIA-certified laboratory for the routine management of SCCA, we performed a retrospective, single-institution study to correlate serum CEA levels with clinical and pathologic outcomes in patients across all stages and presentations of SCCA.

## RESULTS

### Clinical characteristics

The clinicopathologic characteristics of the patients in this retrospective study are summarized in Table 1. The median age of patients was 56 years (interquartile range, 49–61). The majority of patients analyzed were female (74%) and of Caucasian ethnicity (89%). The most common stage for SCCA at initial presentation was stage III (39%). For the 219 patients with biopsy-proven SCCA at the time of initial CEA measurement at our institution, 39 (18%) were newly diagnosed/non-metastatic, 17 (8%) with no evidence of disease following chemoradiation, 16 (7%) patients with recurrent/resectable disease, 19 (9%) with locally advanced/unresectable disease, 17 (8%) with metastatic disease to lymph nodes only, and 91 (41%) with metastatic disease to distant organs at the time of the initial CEA measurement. For the 146 tumors with known HPV status, 140 (96%) were confirmed HPV-positive, and 6 (4%) were HPV-negative. The majority of patients (52%) with available tobacco history had no prior exposure to tobacco.

**Table 1:** Demographic and clinical characteristics of participants with SCCA

Gender	Total *N*	%
Female	162	74.0
Male	57	26.0
Race/ethnicity		
White	195	89.0
Black	12	5.5
Hispanic	6	6.0
Other	6	6.0
Age at Initial Diagnosis		Range
Mean (SD), years	55.5 (9.6)	31–84
Stage at Initial Diagnosis		
I	14	6.56
II	53	24.9
III	83	39.0
IV	63	29.6
HPV Status by *in situ* hybridization		
Negative	29	13.2
Positive	80	36.5
Not Available	110	50.2
P16 Status		
Negative	11	5.1
Positive	126	58.1
Not available	80	36.9
HPV Status (Combined)		
Negative	6	2.7
Positive	140	63.9
Not Available	73	33.3
CEA Level (ng/mL)		Range
Mean (SD)	10.8 (69.2)	0.1–970
Clinical Presentation		
D (newly diagnosed/non-metastatic)	39	17.8
M-L (metastatic SCCA with distant lymph node-only involvement)	17	7.8
M-V (metastatic SCCA to distant, visceral organs (“M-D”))	91	41.2
N (no evident residual disease)	17	7.8
R (recurrent following definitive chemoradiation/resectable SCCA)	16	7.3
U (recurrent following definitive chemoradiation/unresectable SCCA)	19	8.7
Not Available	20	9.1
HIV Status		
Negative	210	95.9
Positive	8	3.7
Not available	1	0.5
History of Tobacco Exposure		
Absent	114	52.1
Present	101	46.1
CEA Category (adjusted for current smoking status)		
Normal	156	72.6
Elevated	59	27.4

### Mean CEA at time of presentation

**Figure 1:**
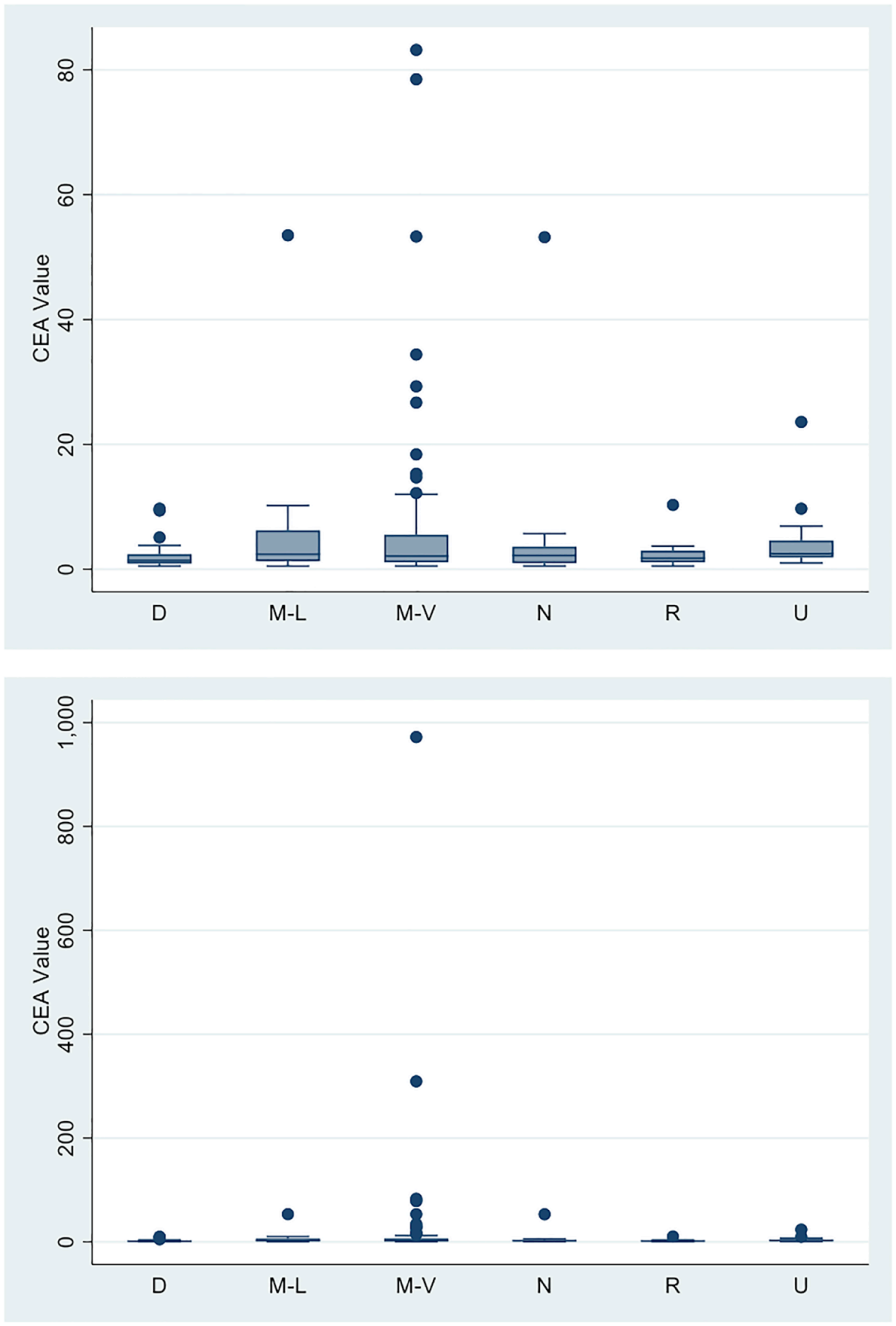
Mean CEA values according to clinical presentation.

The mean CEA level of our patients was 10.76 (SD: 69.2). Figure 1 shows the mean CEA level by clinical status at the time of presentation to our institution.

**Table:** 

Clinical presentation at time of CEA collection	Mean CEA	SD	Range
Newly diagnosed/non-metastatic SCCA (D)	2.1	2.1	0.5–9.7
Metastatic SCCA with distant lymph node-only involvement (M-L)	6.5	12.5	0.5–53.5
Metastatic SCCA to only non-lymph node distant organs (M-V)	20.7	106.6	0.5–972
No evidence of recurrent/residual SCCA (N)	5.4	12.4	0.5–53.2
Recurrent/resectable SCCA following definitive chemoradiation (R)	2.4	2.4	0.5–10.3
Recurrent /unresectable SCCA following definitive chemoradiation (U)	4.4	5.2	1–23.6

The mean CEA at the time of initial diagnosis for locoregional SCCA prior to initiation of chemoradiation was 2.1 ng/mL, whereas patients finishing chemoradiation with no evidence of disease had mean levels of 5.4 ng/mL. The mean CEA of patients with metastatic disease to distant organs was 20.7 ng/mL. CEA levels in all other clinical subgroups were lower, ranging from 2.4 ng/mL in the group of patients in the group of patients with recurrent/resectable disease, to 6.5 ng/mL (interquartile range (IQR), 1.3–6.2 ng/mL) in the group of patients with metastases to distant lymph nodes only. No appreciable differences in mean CEA levels according to disease status were detected by ANOVA (*p* = .74).

We further classified CEA values at presentation into high vs low depending on the individual’s smoking status. i.e., if an individual was a smoker and had a CEA value of ≥ 5, it was classified as high. For non-smokers we classified CEA ≥ 3 as high.

**Figure 2:**
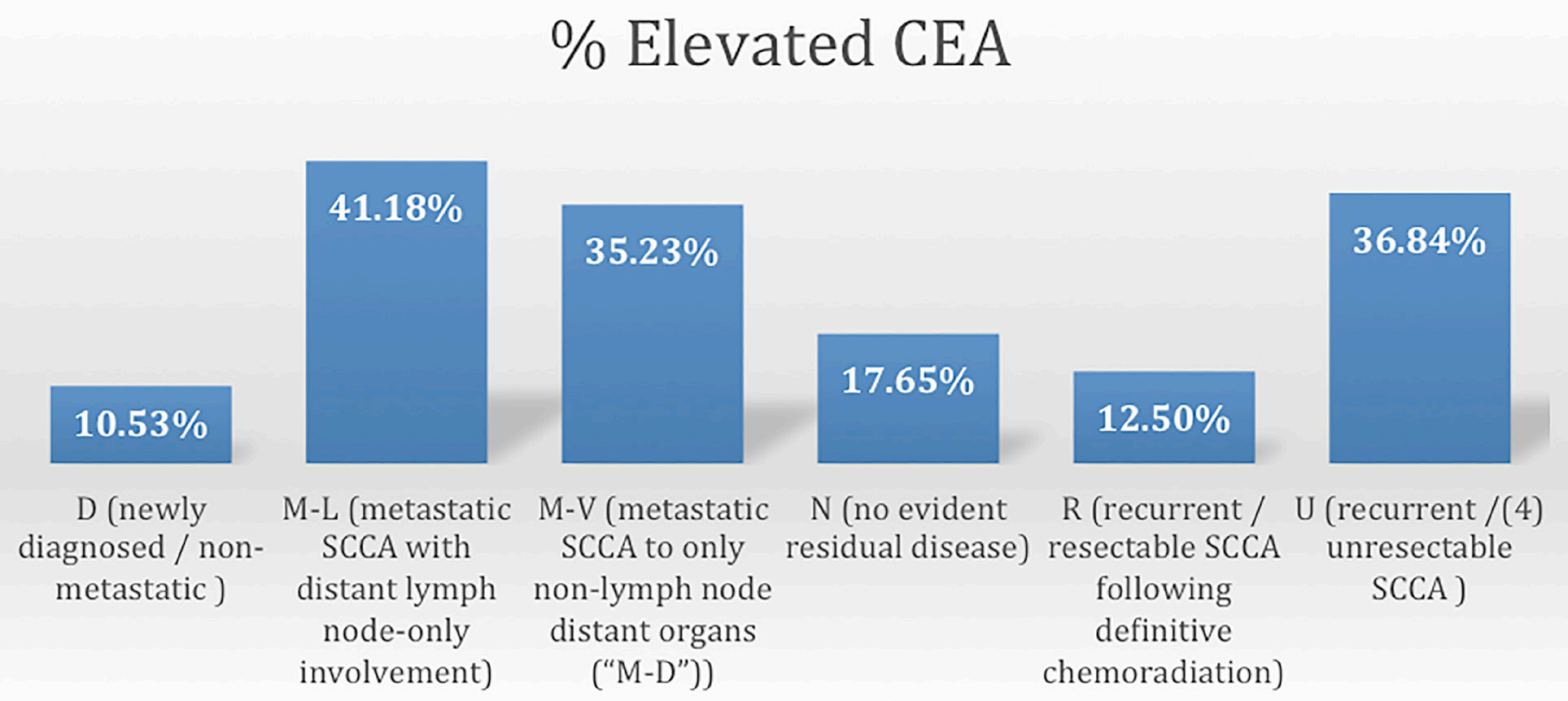
Frequency of elevated CEA according to disease status of SCCA.

As seen in Figure 2, patients with elevated CEA differed according to the clinical status at presentation (Fisher’s Exact test = 0.017). Relative to patients with newly diagnosed, nonmetastatic SCCA (11%), abnormally elevated CEA values occurred more frequently in patients with unresectable/incurable disease (37%) with distant lymph node metastases (42%), and with distant visceral organ involvement (35%).

### Correlating change in CEA with change in radiographic tumor burden

For those patients with distant metastatic disease with serial CEA values drawn while receiving cytotoxic chemotherapy, we found that for 35 participants with documented progression, the mean change in CEA values for patients with documented tumor progression was an increase of 19 ng/ml (range: 12.2 to 176.5 ng/ml), whereas for 23 participants with documented interval disease response, the mean change in CEA values was −7.3 (range: −82.4 to 2.4). Here, average changes in CEA differed between patients who had reductions versus increases in tumor sizes while on cytotoxic chemotherapy (*p* = 0.004).

### Serum CEA level as a prognostic biomarker in metastatic SCCA

The median survival of patients with distant metastatic diseases was 12.5 months (range: 1–55 months). We assessed whether or not CEA levels were associated with survival outcomes for all patients with incurable, metastatic SCCA. Here, CEA levels were assessed as a continuous variable in monitoring for any association with survival (Table 2). CEA level was a weak predictor for death, with a CEA level of 8 showing the highest AUC (0.59) on ROC analysis (Table 2A). Even so, this association was not significant, and AUC values did not appreciably vary between various CEA cutoff levels between 1–10. As show in in Table 2B, correction for age in a multivariate analysis did not affect the inability of CEA level to predict the likelihood for survival outcome in patients with metastatic SCCA.

**Table 2A:** CEA value ROC curve for predicting death for patients with metastatic disease

CEA cutoff (ng/mL)	Parametric			Non-Parametric		
	ROC	Lower CI	Upper CI	ROC	Lower CI	Upper CI
1	0.5229	0.44398	0.60182	.5228994	.4467061	.5990927
2	0.5712	0.47603	0.66642	.5712225	.4776057	.6648393
3	0.5200	0.42433	0.61570	.5200144	.4234034	.6166254
4	0.5029	0.41006	0.59571	.502885	.4083867	.5973833
5	0.5261	0.43623	0.61606	.526145	.4362191	.6160708
6	0.5494	0.46349	0.63532	.549405	.4638112	.6349988
7	0.5727	0.49200	0.65333	.572665	.4923749	.6529551
8	0.5896	0.51153	0.66770	.5896141	.4933023	.6390454
9	0.5662	0.49368	0.63867	.5661738	.4933023	.6390454
10	0.5449	0.47601	0.61378	.5448972	.4752871	.6145074

**Table 2B:** Hazard ratio for metastatic SCCA at different CEA cut-offs

CEA cutoff (ng/mL)	Univariate			Multivariate (Age adjusted)		
	HR	Lower CI	Upper CI	HR	Lower CI	Upper CI
1	1.43	0.67	3.08	1.44	0.67	3.10
2	0.97	0.53	1.80	0.97	0.52	1.78
3	1.04	0.57	1.88	1.04	0.97	1.03
4	1.07	0.58	1.98	1.07	0.57	2.02
5	1.07	0.57	2.02	1.08	0.57	2.07
6	1.10	0.58	2.11	1.11	0.58	2.14
7	1.01	0.52	1.94	1.91	0.52	1.96
8	1.10	0.58	2.11	1.01	0.52	1.96
9	0.86	0.42	1.78	0.86	0.42	1.80
10	1.32	0.63	2.77	1.32	0.63	2.77

### Relationship between tumor IHC stain for CEA and serum CEA

Sixteen formalin-fixed paraffin-embedded tumor samples from patients with metastatic SCCA were stained by IHC for CEA expression. Of these 16 samples, 6 had tumor staining for CEA with 3+ intensity by IHC in > 40% of tumor cells. All 6 of these with high tumor expression of CEA had abnormally elevated CEA levels. For those banked samples with CEA staining < 40% or with no CEA staining (*n* = 10), 9 samples corresponded to normal serum CEA levels, and 1 sample had an abnormally elevated CEA. There was an association between CEA expression by immunohistochemistry and corresponding serum CEA levels (OR = 82, *p* < 0.01).

## DISCUSSION

Here we report the largest series detailing the relevance of CEA as a biomarker for patients with SCCA. Elevated CEA levels (30% of all patients in our cohort) occurred more commonly in more advanced stages of disease. However, the majority of patients with metastatic SCCA did not have elevated CEA levels in the serum. While CEA is commonly used in the clinical management of colorectal adenocarcinomas, our data suggest that its applicability does not extend reliably to the clinical management of patients with squamous cell malignancies affecting the adjacent anal canal.

Patients with metastatic anal cancer more frequently have abnormally high serum CEA levels relative to patients with newly diagnosed nonmetastatic disease or without evidence of disease following completion of chemoradiation. Our study population is relevant given that our patients’ demographics match similarly to those historically reported in the United States with anal cancer – i.e., more common in females than in males and over the age of 50 [13–15]. We detected abnormally high CEA levels in approximately 40% of metastatic SCCA involving distant visceral organs (e.g., liver, lung, and/or bone) but also in patients with distant non-regional lymph node involvement only. Interestingly, despite the similar prevalence of abnormal CEA levels in these two metastatic groups, mean CEA levels trended higher (22 ng/mL vs. 6 ng/mL) in patients with distant organ metastases. Therefore, the applicability of CEA monitoring may be perhaps best reserved for this subpopulation of patients with metastatic SCCA.

One previous series examined CEA levels in SCCA patients, and likewise found no relationship between serum CEA level and clinical or pathologic features [12]. This series was limited, however, to patients with non-metastatic disease undergoing definitive chemoradiation with curative intent. For this population in our study, serum CEA levels were likewise not elevated in general. Here, CEA expression on the tumor cell surface was detected in a small fraction (15%) of patients with newly diagnosed, locoregional SCCA treated with curative intent therapies. Our data regarding the relative lack of utility for serum CEA in the management of SCCA are strengthened by the inclusion of patients across all stages of disease presentation.

For patients with metastatic SCCA, we observed no association between survival and serum CEA level at the time of presentation of initial diagnosis of distant metastases. For patients with resected adenocarcinoma of the colon or rectum, a CEA cut off ≥ 10 ng/mL was linked in a pooled meta-analysis with an ability for eventual detection of recurrent disease prior to radiographic appearance with a sensitivity of 68% and a specificity of 97% [16]. In that study, for colorectal cancer an elevated CEA served as a harbinger for a poor clinical outcome and served as a surrogate for microscopic residual disease. Here we assessed CEA as a biomarker for survival outcomes when patients had higher tumor burdens present in the setting of macroscopic, radiographically evident metastatic SCCA. Even with a cutoff for CEA at 10 ng/mL, we did not predict survival for patients with metastatic SCCA. Even though, when elevated, trends in relative levels over time did appear to reflect the associated radiographic response to systemic therapy, the relative paucity of elevated CEA levels in this cohort renders this assay clinically unreliable in the management of metastatic SCCA.

Currently there is a need for improving upon anti-PD-1 antibodies as monotherapy as an immunotherapy backbone for patients with unresectable, incurable SCCA. A phase II trial with single-agent nivolumab demonstrated a response in 24% of patients, [7] and another single-arm study of patients with metastatic SCCA treated with pembrolizumab revealed a response rate of 10% in over 100 patients [8]. However, despite initial promise with single-agent anti-PD1 therapy, the majority of patients do not benefit for these agents, highlighting an opportunity for further combination immunotherapy regimens. Interestingly, our study demonstrates an association between expression of CEA protein on tumor cells with corresponding serum CEA levels, when elevated, in patients with metastatic SCCA.

A recent study of cibisatamab, a bifunctional CD3^+^ T cell-CEA bispecific antibody able to introduce immunoreactive T-cells with CEA-expressing tumor cells, in combination with the anti-PD-L1 antibody atezolizumab reported promising (though early) disease control rate in patients with microsatellite stable metastatic colorectal cancer, [17] a population which does not respond commonly to antiPD-1/ anti-PD-L1 therapies [18]. Given the reported activity of such agents in a tumor type with frequent overexpression of CEA on tumor cells, our findings provide rationale for testing CEA-targeted immunotherapies in patients with metastatic SCCA. Here, a serum CEA test could serve as an inexpensive tool for screening of CEA cell surface expression. We acknowledge several limitations to our retrospective study. First, no differences in mean CEA levels were significant between the various groups of patients with SCCA analyzed. However, this could be due to low sample sizes among all subgroups for this rare malignancy, as outlier results (e.g., > 100 ng/mL) increased the standard error calculations in our statistical analysis. Nonetheless, the mean CEA levels trended higher with advanced disease and were confirmed more frequent in this subpopulation. In addition, CEA is a non-specific marker that may be elevated in non-malignant and other malignant conditions as well. While use of a more specific HPV circulating tumor DNA assay has demonstrated early promise for predicting recurrence in a research-laboratory setting [10, 11], our findings capitalize upon a well-validated assay performed in a CLIA-certified setting and readily available to oncologists in academic and community settings alike. We foresee that ctDNA assays identifying HPV-specific oncogenes may become available as a more reliable biomarker for response to treatments for SCCA in the future.

In summary, we report the largest series to describe CEA as a serum biomarker for patients with metastatic SCCA. Our findings may not provide definitive support for the use of a routinely used blood-based assay for management of patients with SCCA and should guide clinicians in seeking alternative approaches for tracking responses to treatment in this disease. Nonetheless, novel approaches with serum biomarkers are needed for patients with this rare but increasingly diagnosed malignancy.

## MATERIALS AND METHODS

Under an IRB-approved protocol at our institution, an electronic database of medical records from 219 patients with pathologically confirmed SCCA who were evaluated and treated at MD Anderson Cancer Center between 2013–2020 was retrospectively reviewed in order to collect demographic data, clinical history and CEA levels. Baseline characteristics collected and analyzed included gender, ethnicity, stage at initial diagnosis of SCCA, HPV status, HIV status and smoking history. No patients with coexisting second primary cancers besides SCCA were included in this analysis. Of these 219 patients with SCCA, 119 patients had more than 1 CEA level available. This subset of patients was analyzed further for an association between clinical outcome in relation to changes in CEA level. HPV status was classified as “HPV-positive” if detected according to one of two methods: (1) measurement of HPV DNA in tumor tissue by *in situ* hybridization for HPV (PathoGene HPV type 16/18/33/51 probe; Enzo Life Sciences, Inc., Farmingdale, NY), using a method previously described or (2) detection of the p16 protein by immunohistochemistry [19]. For correlations with associated CEA levels (ng/mL), patients were categorized into 6 separate clinical scenarios: (1) newly diagnosed/non-metastatic (“D”); (2) with no evident residual disease following definitive chemoradiation (“N”); (3) recurrent/resectable SCCA following definitive chemoradiation (“R”); (4) recurrent/unresectable SCCA (“U”); (5) metastatic SCCA with distant lymph node-only involvement (“M-LN”); and (6) metastatic SCCA with distant, visceral organ involvement (“M-D”). Patients with metastatic disease to both distant organs and to distant lymph nodes alike were classified as “M-D”. An abnormal CEA was defined based on institutional practices as a CEA > 3 ng/mL for non-smokers, and > 5 ng/mL for current smokers. Our study also examined available banked primary or metastatic tumor specimens (2 primary, 6 recurrent/resected and 8 metastatic tumors) from 16 matched patients available after January 2010 for CEA expression with immunohistochemical staining for CEA using a monoclonal antibody against CEA (AB-2 clone Lab Vision/NeoMarkers, Freemont, CA) at a 1:200 dilution. An association between tumor expression of CEA by immunohistochemistry and corresponding serum CEA level obtained at the same time point was tested using a Fisher’s exact *t*-test.

Demographic and clinical characteristics of patients were summarized as means with associated standard deviations (SD) for continuous variables and as events (N) with associated frequencies (%) for categorical variables. Mean CEA levels were compared between all six subgroups via ANOVA analysis (SPSS, Armonk, NY, USA). Frequencies of elevated CEA between all six subgroups were compared via Chi-squared test. For patients with distant metastases, “metastatic survival” was defined as the date of diagnosis of metastatic disease until the date of last follow up or death. Patients lost to follow up were censored at their last follow up visit. Mean metastatic survival was estimated for patients with high CEA (CEA ≥ 10 ng/mL) and low CEA (CEA < 10 ng/mL) using Kaplan-Meier analysis and compared via log-rank test. Hazard ratios were estimated with univariate Cox proportional hazard models using Prism 7 software (GraphPad, La Jolla, CA, USA). All *p*-values presented are two-sided. We further assessed the discriminant validity of CEA as a predictor of survival by measuring area under the curve using receiver operator curve. The ROC curves help to measure how well our cut-off for CEA can help discriminate or help determine survival status of patients with metastatic SCCA. It provides with an estimate of area under the curve which ranges from 0 to 1 with higher scores indicating high performance as a distinguishing variable. An AUC value of 0.9–1 indicates very good distinguishing ability, 0.8–0.9 indicates good discriminant ability, a score of 0.7 to 0.8 indicates fair discriminant ability, a score of 0.7 and below indicates poor discriminant ability.

## Abbreviations

SCCA: squamous cell carcinoma of anus
CEA: carcinoembryonic antigen
ctDNA: circulating tumor DNA
SD: standard deviation
